# Association between work productivity and characteristics of adults with X-linked hypophosphatemia: an analysis of the XLH disease monitoring program

**DOI:** 10.1093/jbmrpl/ziae102

**Published:** 2024-07-29

**Authors:** Aliya Khan, Ben Johnson, Annabel Nixon, Jennifer E Dent, Zhiyi Li, Erru Yang, Angela Williams

**Affiliations:** McMaster University, Hamilton, Ontario, L8N 3Z5, Canada; Health Economics and Outcomes Research, Kyowa Kirin International, Marlow SL7 1HZ, United Kingdom; Patient Centered Outcomes, Chilli Consultancy, Salisbury SP1 1JS, United Kingdom; Patient Centered Outcomes, Chilli Consultancy, Salisbury SP1 1JS, United Kingdom; Health Economics and Outcomes Research, Kyowa Kirin North America, Bedminster NJ 07921, United States; Global Health Economics and Outcomes Research, Ultragenyx Pharmaceutical Inc., Novato, CA 94949, United States; Health Economics and Outcomes Research, Kyowa Kirin International, Marlow SL7 1HZ, United Kingdom

**Keywords:** X-linked hypophosphatemia (XLH), work productivity, disease monitoring program, patient-reported outcomes, FGF 23

## Abstract

X-linked hypophosphatemia (XLH) is a rare, genetic, progressive, phosphate-wasting disorder that causes skeletal morbidities, stiffness, pain, and impaired physical function. This study used baseline data from the XLH Disease Monitoring Program to evaluate relationships between work productivity and patient characteristics (demographics, medical history, patient-reported, and functional outcomes) in adults with XLH. Bivariate analysis guided the selection of variables for multivariate analysis after adjustment for multicollinearity and conceptual overlap. The analysis comprised 281 subjects (75.4% female; 80.8% from USA; median age 39.2 yr); 53.4% were employed full-time and 31.3% were not employed; 15.3% were receiving disability payments; 47.0% were taking burosumab at study entry. Most employed subjects were working full-time outside the home (69.9%) and in light or sedentary roles (59.6%). In multivariate analyses, patients with fewer orthopedic surgeries (odds ratio [OR] 0.88; 95% confidence interval [CI], 0.81–0.96; *p*=.002) and better Patient-Reported Outcomes Measurement Information System Physical Function scores (OR 1.08; 95% CI, 1.02–1.15; *p*=.013) were more likely to be in full-time employment than not employed. Younger patients (OR 0.97; 95% CI, 0.94–0.99; *p*=.014) and those with fewer orthopedic surgeries (OR 0.83; 95% CI, 0.73–0.95; *p*=.008) were more likely to be in medium than light or sedentary work. Those with worse WOMAC Pain scores were more likely to be doing heavy/very heavy than light or sedentary activity (OR 1.04; 95% CI, 1.01–1.07; *p*=.006). Full-time employment levels are low in adults of working age with XLH, and unemployment and disability payment rates are high, suggesting that XLH has a substantial impact on work productivity. Worse physical function and a greater number of orthopedic surgeries are associated with lower work productivity. Worse pain, higher number of orthopedic surgeries, and younger age are associated with heavier work roles; however, causality was not specifically investigated.

## Introduction

X-linked hypophosphatemia (XLH) is a rare, genetic, progressive, phosphate-wasting disorder that is caused by loss-of-function mutations in the *PHEX* (phosphate-regulating endopeptidase homologue, X-linked) gene. The resulting excess circulating levels of fibroblast growth factor 23 (FGF23) lead to renal phosphate wasting and hypophosphatemia,^1^ with deleterious effects on bone growth and quality (bone turnover, microarchitecture, and mineralization), abnormalities in muscle structure and function, and impaired dental mineralization.^1–4^

XLH typically manifests in childhood as rickets, skeletal deformities, and short stature, as well as dental complications including abscesses.^5–7^ These impairments persist into adulthood, compromising physical function.^8^ Further morbidities develop in adulthood, including osteoarthritis, fractures and pseudofractures, osteophytes, enthesopathy, and spinal stenosis, which can cause further impairments.^8^^,^^9^ Patients also frequently require corrective surgery for skeletal deformities, including osteotomy, hip and knee arthroplasty, spinal surgery, and surgical fixation of fractures.^8^^,^^9^ XLH is associated with impaired physical function, stiffness, and pain^10^; patients report notable difficulty and pain going up and down stairs and doing heavy household chores, and stiffness on waking. Mean 6-min walk test distance is approximately half of the predicted distance based on age and sex.^10^ XLH is also associated with impaired mental health, including depression, anxiety, distress, frustration, low self-esteem, worries about the future, difficulties in intimate relationships, and social isolation.^11–13^

Chronic conditions can compromise patients’ ability to engage in and maintain work at full capacity^14^ and contribute to the economy. Work productivity is increasingly evaluated as a patient-centered outcome and is an important element of economic analyses that adopt a societal perspective.^14^ Decreased on-the-job productivity and employee absence because of health issues can lead to significant costs to employers that may exceed direct medical expenditures.^15^ Research exploring associations between health utility and work productivity in people living with chronic and severe diseases indicates that better health states are expected to be associated with higher productivity.^16^ However, the impact of functional impairment in XLH on work productivity has not been documented.

A disease monitoring program (DMP) provides an alternative to a traditional registry and extensive post-marketing studies, enabling enhanced understanding of a rare disease and its response to treatments over time.^17^ DMPs use a collaborative, multi-stakeholder approach to monitor disease manifestations over an extended period, without limiting participants based on treatment received. The XLH Disease Monitoring Program (XLH-DMP^18^) provides a large dataset (>400 adults), which we have used to explore work productivity in adults with XLH.

### Objective

The objective of this study is to understand the impact of XLH on work productivity and to assess the association between patient characteristics (demographics, medical history, and patient-reported and functional outcomes) and work productivity, using data from the XLH-DMP.

## Materials and methods

### Study data

The XLH-DMP is an international, prospective, 10-yr, longitudinal, observational study of adults and children with XLH (NCT03651505). It was established in response to a request by the Food and Drug Administration (FDA) to follow maternal and safety outcomes, using a public–private partnership model to ensure consistent collection, ownership sharing, and governance of data.^17^ The steering committee includes clinical experts in XLH, patient advocates from the XLH Network, and representatives from Ultragenyx Pharmaceutical and Kyowa Kirin. The study has been collecting demographic, biochemical, clinical, disease severity, patient-reported outcomes (PROs), treatment, and progression data since 2018. The XLH-DMP population comprises adults and children with XLH in the USA, Argentina, Brazil, Canada, Chile, and Colombia, who may or may not be receiving treatment with burosumab or other treatment options at study baseline or at any point in the study. To be included in the program, subjects must have a clinical diagnosis of XLH based on family history, confirmed *PHEX* mutation, or a biochemical profile consistent with XLH. Some subjects entered the program from clinical trials. To maximize recruitment, few exclusion criteria were applied; only individuals concurrently enrolled in an Ultragenyx-sponsored clinical trial, those with serious medical or psychiatric comorbidities or life expectancy of less than 1 yr were excluded. Subjects in the XLH-DMP may receive treatment with burosumab (a monoclonal antibody that binds to and inhibits the activity of FGF23) through authorized prescription but treatment is not provided via the program.

The current analysis included adults of working age in the XLH-DMP (≥18 to <65 yr at enrolment) who were enrolled before December 31, 2019. Participants who enrolled after this date were excluded to avoid confounding effects of the COVID-19 pandemic on work productivity.

### Data collection

Patient characteristics and work productivity data were obtained from the XLH-DMP at baseline (ie on inclusion in the program). Patient characteristics comprised demographics, medical history, and patient-reported and functional outcomes: the Western Ontario McMaster Universities Osteoarthritis (WOMAC®) Index, the Patient-Reported Outcomes Measurement Information System (PROMIS) Physical Function score, and the Timed Up and Go (TUG) assessment. The WOMAC Index provides information on pain, stiffness, and physical function^19^ and has been validated for use in XLH.^20^ PROMIS Physical Function is designed to assess the degree of difficulty in completing 9 activities of daily living in individuals with musculoskeletal disorders. The TUG assessment records the time taken (in s) to rise from a chair, walk 3 m, return to the chair, and sit down again.

Three aspects of work productivity were recorded: work status (full-time, part-time, or not employed); employment description (full-time inside the home, full-time outside the home, part-time inside the home, and part-time outside the home); and work activity level (light/sedentary, medium, and heavy/very heavy).

### Analyses

The Pain (5 items), Stiffness (2 items), and Physical Function (17 items) domains of the WOMAC Index can be combined to give a total score.^19^ For ease of interpretation, domain and total scores are normalized to a scale of 0–100, where 0 is the best health state and 100 the worst. PROMIS Physical Function score items are combined using a T-score metric calibrated to a general US reference population with a mean of 50 and SD of 10. Lower scores indicate greater detriment.^21^

WOMAC Index scores were also mapped to EuroQol 5-dimension 3-level (EQ-5D-3L) utility values (England value set) using the algorithm developed by Wailoo and colleagues.^22^ A utility value of 1 represents perfect health; 0 represents a health state equivalent to death.

Only baseline data are analyzed (there is no analysis of treatment effect). The analysis descriptively assessed work productivity (work status, employment description, work activity level), demographics, medical history, WOMAC Index domain and total scores, PROMIS Physical Function scores, TUG time, and utility values. Median, first and third quartiles (Q1–Q3), mean, and SD are reported for continuous variables; categorical data are reported as number and percentage of subjects.

Bivariate analyses (general linear models for unbalanced ANOVA and chi-squared test) were used to evaluate relationships between patient characteristics and work status, employment, and work activity level. The results of this analysis guided the selection of variables for multivariate analyses. Variables with *p*≤.1 were selected for inclusion, allowing variables that demonstrated a trend with work productivity to be taken into consideration in the models. Selection of variables for the models also took into consideration issues of multicollinearity, conceptual overlap of fracture variables and PRO variables, and missing data. Multicollinearity was assessed by considering the correlations between potential explanatory variables; variables that were highly correlated (>0.8) were used to guide selection/exclusion from the models.

Multinomial logistic regression models were used to assess relationships between work productivity (work status, employment description, and work activity level) and patient characteristics (demographics, medical history, and patient-reported and functional outcomes). In the primary analysis, all patient-reported and functional outcomes (WOMAC Index domain scores, PROMIS Physical Function scores, and TUG time) were potential candidates for inclusion in multivariate analysis, with the exception of EQ-5D utility values as these were derived directly from WOMAC scores. In a secondary analysis, multinomial logistic regression models were repeated using utility values but excluding all other patient-reported and functional outcomes. In addition, bivariate logistic regression models were produced for utility values against work productivity.

## Results

### Demographics and medical history

The analysis comprised 281 subjects, 75.4% of whom were female, with a median (Q1–Q3) age of 39.2 (30.4–49.0) yr (Table 1). The median age at XLH diagnosis was 2 (1–6) yr. Most subjects (80.8%) were from the USA. Just fewer than half of the subjects were taking burosumab at study entry (47.0%), and 26.7% were burosumab naïve. Demographic, medical history, and burosumab treatment are reported in Table 1.

**Table 1:** Demographics and medical history (*n* = 281).

**Subject characteristic**			**Value** ^**a**^
**Age (yr) (*n* = 281)**	Mean ± SD		39.9 ± 12.3
	Median (Q1–Q3)		39.2 (30.4–49.0)
**Sex (female), *n* (%) (*n* = 281)**			212 (75.4)
**Country^a^, *n* (%) (*n* = 281)**	USA		227 (80.8)
	Brazil		24 (8.5)
	Canada		20 (7.1)
	Chile		10 (3.6)
**Age at XLH diagnosis, years (*n* = 281)**	Mean ± SD		7.7 ± 13.0
	Median (Q1–Q3)		2.0 (1.0–6.0)
**Any family members with XLH, *n* (%) (*n* = 281)**			215 (76.5)
**Medical history, *n* (%) (*n* = 281)**	Osteoarthritis		161 (57.3)
	Enthesopathy/ bone spurs/ osteophytes		150 (53.4)
	Hyperparathyroidism		69 (24.6)
	Nephrocalcinosis		49 (17.4)
**Number of orthopedic surgeries per subject (*n* = 280)**	Mean ± SD		3.8 ± 4.7
	Median (Q1–Q3)		2 (0–5)
**Time from most recent orthopedic surgery to DMP enrolment, years (*n* = 202)**	Mean ± SD		15.0 ± 12.5
	Median (Q1–Q3)		11.2 (5.4–21.2)
**Age at first orthopedic surgery, years (*n* = 202)**	Mean ± SD		15.1 ± 10.2
	Median (Q1–Q3)		13.2 (8.4–18.2)
**Age at first fracture/ pseudofracture, years (*n* = 137)**	Mean ± SD		24.8 ± 13.6
	Median (Q1–Q3)		22.9 (13.4–35.3)
**Time since first fracture/pseudofracture to DMP enrolment, years (*n* = 137)**	Mean ± SD		18.2 ± 13.3
	Median (Q1–Q3)		16.4 (7.1–27.7)
	N		
**Number of fractures/ pseudofractures per subject**	All fractures/ pseudofractures (*n* = 277)	Mean ± SD	2.1 ± 5.8
		Median (Q1–Q3)	0 (0–2)
	Lower extremity fractures (*n* = 278)	Mean ± SD	1.6 ± 4.7
		Median (Q1–Q3)	0 (0–2)
	Non-traumatic/ pseudofractures (*n* = 277)	Mean ± SD	1.5 ± 5.2
		Median (Q1–Q3)	0 (0–1)
	Traumatic fractures (*n* = 280)	Mean ± SD	0.6 ± 1.5
		Median (Q1–Q3)	0 (0–1)
**Use of orthoses (*n* = 264), *n* (%)**			19 (6.8)
**Use of assistive devices (*n* = 264), *n* (%)**			13 (4.6)
**Pain and/or stiffness (*n* = 281), *n* (%)**			256 (91.1)
**Timed Up and Go time (s) (*n* = 264)**	Mean ± SD		10.1 ± 6.0
	Median (Q1–Q3)		9.4 (7.8–10.8)
**Disability payment**	Receiving payments, *n* (%) (*n* = 281)		43 (15.3)
	Age at first payment (*n* = 38)	Mean ± SD	35.6 ± 9.8
		Median (Q1–Q3)	35.0 (28.3–41.5)
**Burosumab treatment at study entry, *n* (%) (*n* = 281)**	Taking burosumab		132 (47.0)
	Not taking burosumab		149 (53.0)
**Burosumab exposure, *n* (%) (*n* = 281)**	Previously or currently taking		206 (73.3)
	Never taken		75 (26.7)

a There were no subjects from Argentina or Colombia

### Work productivity

**Figure 1:**
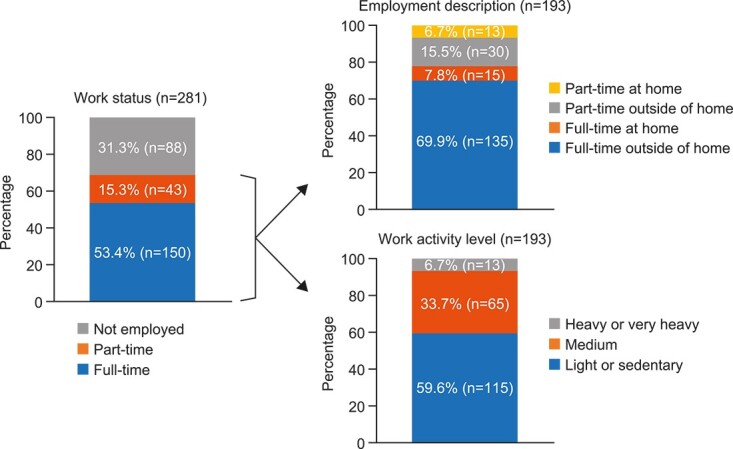
Work productivity (*n* = 281).

Just over half the subjects were employed full-time (53.4%) and just under a third were not employed (31.3%) (Figure 1); 15.3% were receiving disability payments (Table 1). Among the 193 subjects who were employed full- or part-time, most were working full-time outside of home (69.9%) and were in light or sedentary roles (59.6%) with few in a heavy or very heavy role (6.7%).

### Patient-reported and functional outcomes

**Figure 2:**
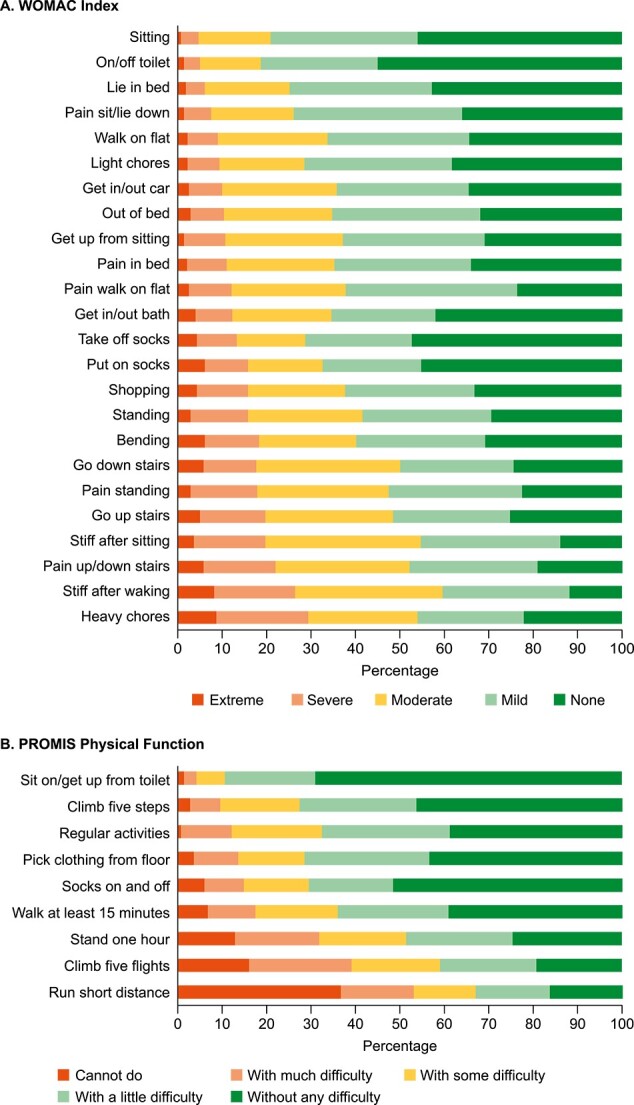
WOMAC Index and PROMIS Physical Function item responses. (a) WOMAC Index. (b) PROMIS Physical Function.

WOMAC Index domain scores were highest (indicating worse symptoms) for Stiffness, followed by Pain and Physical Function (Table 2). As shown in Figure 2a, the greatest impairments were seen in heavy household chores, early morning stiffness, and pain when going up or down stairs (29.4%, 26.4%, and 22.0%, respectively, reported severe/extreme difficulty for the items). EQ-5D utility values ranged from −0.1 to 1.0, with a median (Q1–Q3) of 0.750 (0.588–0.900) (*n* = 277).

**Table 2:** WOMAC index and PROMIS Physical Function scores.

**PRO**	**Domain**	***n***	**Mean ± SD**	**Median (Q1**–**Q3)**	**Range**
**WOMAC Index**	Pain	280	32.5 ± 22.5	30.0 (15.0–45.0)	0.0–100.0
	Stiffness	280	43.3 ± 24.8	37.5 (25.0–62.5)	0.0–100.0
	Physical function	277	29.1 ± 22.8	26.5 (10.3–45.6)	0.0–100.0
	Total score	277	31.0 ± 22.0	28.1 (12.5–47.9)	0.0–100.0
	EQ-5D utility value	277	0.701 ± 0.229	0.750 (0.588–0.900)	−0.1 to 1.0
**PROMIS**	Physical function	281	41.6 ± 8.8	40.7 (35.9–46.9)	15.5–60.3

WOMAC Index domain and total scores are normalized to 0–100, where 0 is the best health state and 100 the worst.

PROMIS uses a T-score metric calibrated to a reference population with a mean of 50 and SD of 10. Higher scores indicate better physical function.

Utility values less than 0 represent health states regarded as worse than death.

For PROMIS Physical Function, the greatest difficulty related to running short distances, climbing 5 flights of stairs, and being able to stand for an hour (53.1%, 39.1% and 31.8%, respectively, responded cannot do/can do with much difficulty for these items; Figure 2b).

The median (Q1-Q3) TUG time was 9.4 s (7.8–10.8).

### Relationships between work productivity and patient characteristics

#### Work status

In the bivariate analysis, work status (ie employed full-time or part-time or not employed) was significantly related to study country (*p*=.019) (Table S1). In terms of XLH characteristics, work status was significantly related to history of osteoarthritis (*p*=.017); number of orthopedic surgeries (*p*<.001); age at first fracture (*p*=.043); numbers of fractures (*p*=.002), lower extremity fractures (*p*=.004), non-traumatic/pseudofractures (*p*=.006), and traumatic fractures (*p*=.034) (Table S2). In terms of functional and patient-reported outcomes, work status was significantly related to use of assistive devices (*p*=.005), TUG time (*p*=.002), PROMIS Physical Function score (*p*<.001), WOMAC Pain (*p*=.006), Physical Function (*p*=.009) and total score (*p*=.011), EQ-5D utility value (*p*=.002), and receiving disability payments (*p*<.001) (Table S3).

Multicollinearity (≥0.8) was identified between WOMAC Physical Function and total scores (0.99); WOMAC Pain and total scores (0.93); WOMAC Pain and Physical Function scores (0.88); and WOMAC and PROMIS Physical Function scores (−0.81).

**Figure 3:**
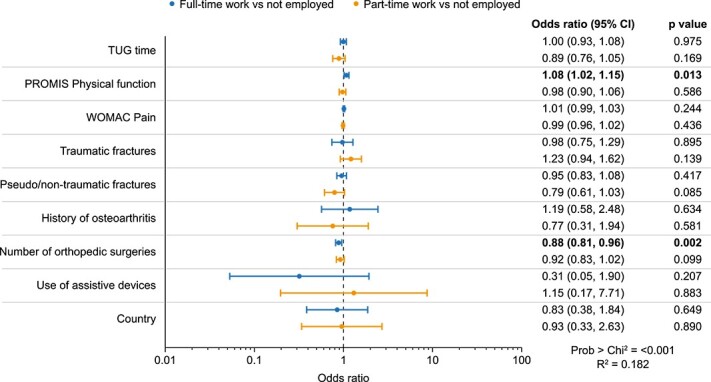
Relationships between work status and patient characteristics (*n* = 260).

WOMAC Pain and PROMIS Physical Function were included in the multivariate analysis because this allowed inclusion of both pain and physical function in the models without issues of multicollinearity. After taking into account multicollinearity and missing data, 9 independent variables were included in the multinomial logistic regression model (Figure 3). The number of orthopedic surgeries and PROMIS Physical Function score was significantly associated with work status: subjects with fewer surgeries (odds ratio [OR] 0.88; 95% CI, 0.81–0.96; *p*=.002) and those with better PROMIS Physical Function scores (OR 1.08; 95% CI, 1.02–1.15; *p*=.013) were more likely to be in full-time employment than not employed.

#### Employment description

In the bivariate analysis, employment description (full- or part-time, inside or outside the home) was significantly related to age (*p*=.040) (Table S1), osteoarthritis (*p*=.033), burosumab treatment at study entry (*p*=.049), age at first fracture (*p*=.010) (Table S2), and receiving disability payments (*p*=.001) (Table S3). There was no multicollinearity among the variables eligible for inclusion in the model. Three independent variables were included in the multinomial model after accounting for missing data, none of which were significant in the model (Figure S1).

#### Work activity level

In the bivariate analysis, work activity level was significantly related to the number of orthopedic surgeries (*p*=.024) (Table S2), WOMAC Pain score (*p*=.027), and EQ-5D utility value (*p*=.013) (Table S3). Four independent variables were eligible for inclusion in the multinomial model based on the bivariate analysis: age, number of orthopedic surgeries, number of non-traumatic/pseudofractures, and WOMAC Pain score. There was no multicollinearity among these.

**Figure 4:**
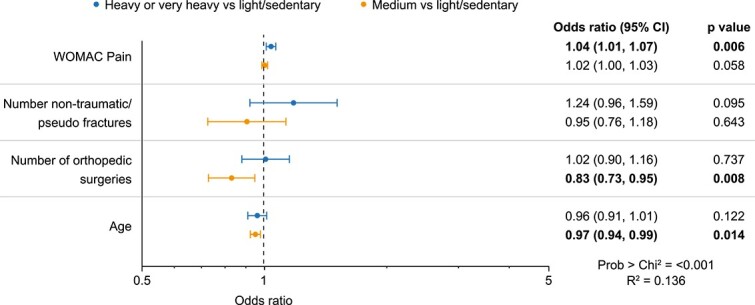
Relationships between work activity level and patient characteristics (*n* = 190).

In multivariate analysis, age, number of orthopedic surgeries, and WOMAC pain score were significantly associated with work activity level (Figure 4): subjects who were younger (OR 0.97; 95% CI, 0.94–0.99; *p*=.014) and those who had undergone fewer orthopedic surgeries (OR 0.83; 95% CI, 0.73–0.95; *p*=.008) were more likely to be in medium activity work than in light or sedentary work. Those with worse WOMAC Pain scores were more likely to be employed in work that involved heavy or very heavy activity than in light or sedentary activity (OR 1.04; 95% CI, 1.01–1.07; *p*=.006).

#### Secondary analysis: EQ-5D utility values included in multivariate models

In the bivariate analysis, EQ-5D utility value was significantly related to work status (*p*=.002) and activity level associated with work (*p*=.013), but not with employment description (*p*=.081) (Table S3), and was eligible for inclusion in all 3 models (ie *p*≤.1). There was no multicollinearity between utility values and other variables included in the models. Seven independent variables were included in the model for work status, 3 in the model for employment description and 4 in the model for work activity level. Utility value was not significant in the multivariate analysis of work status (Figure S2) or employment description (Figure S3). However, utility value was significant in the multinomial analysis of work activity level (Figure S4); those doing heavy or very heavy work activities had lower values than those doing light or sedentary activities (OR 0.02; 95% CI, 0.00–0.25; *p*=.002).

In the bivariate logistic regression models, EQ-5D utility value was significantly related to work status, employment description, and work activity levels (Table S4). Those in full-time employment had higher utility values than those who were not employed (OR 8.14; 95% CI, 2.49–26.60; *p*=.001), part-time work at home was associated with lower values than full-time work outside of the home (OR 0.07; 95% CI, 0.01–0.65; *p*=.020), and heavy or very heavy work activity was associated with lower values than light or sedentary roles (OR 0.04; 95% CI, 0.01–0.42; *p*=.007).

## Discussion

Relationships between health and productivity are receiving increasing attention in business and scientific communities,^23^ and work productivity is an important element of heath economic analysis in chronic diseases. The long-term sequelae and functional impairments associated with XLH might be expected to compromise patients’ work productivity, but this has not been explored to date. The real-world XLH-DMP study provides a unique resource to investigate this.

The current analysis shows that work productivity is compromised in adults of working age with XLH: compared with the US general population, adults with XLH had lower rates of full-time employment (53.4% vs 77.5% [82.5/71.8% in men/women]) and rates of unemployment were 8 times higher (31.3% vs 3.6% [3.7/3.6% in men/women]).^24^ The rate for disability payments was nearly 4 times higher in adults with XLH compared with the general population (15.3% vs 4%).^25^

Many factors affect the ability to work, including age, type of work, and disease-related variables such as pain, swelling, joint stiffness, and functional disability.^26^ The current cohort had medical histories and physical impairments that might be expected to compromise work productivity. More than half had a history of osteoarthritis or enthesopathy (57% and 53%, respectively) and the cohort had undergone an average of 3.9 orthopedic surgeries and had experienced 2.1 fractures or pseudofractures. Subjects also reported substantial impairments in physical function, pain, and stiffness using the WOMAC Index and PROMIS Physical Function instruments. The median PROMIS Physical Function T-score of 40.7 indicates worse physical function than subjects with breast, prostrate, uterine, cervical, colorectal, or non-Hodgkin’s lymphoma cancers, and similar impairment to those with stage 4 cancer.^27^

WOMAC Pain, Physical Function, and Stiffness scores in this real-world cohort indicate less severe impairment than in adults involved in a phase 3 trial of burosumab for the treatment of XLH,^10^ which likely reflects trial inclusion criteria requiring subjects to have a minimum pain score and to be off treatment for trial baseline assessments. WOMAC Pain, Stiffness, and Physical Function scores in the current cohort are closer but less severe than those reported by subjects with XLH in a burden of disease study.^8^ These differences could arise because nearly half the subjects were receiving burosumab treatment at baseline in the XLH-DMP, because the current cohort was restricted to adults of working age and was therefore younger on average, or due to regional differences. The EQ-5D utility value in this study (mean 0.701, median 0.750), derived from WOMAC scores is also higher than published values for patients with XLH based on the EQ-5D-5L (mean 0.648; UK),^28^ the EQ-5D-3L using a crosswalk methodology (mean 0.554, median 0.654; UK),^29^ and the EQ-5D-3L (mean 0.562; Spain).^30^

Multivariate regression analysis showed associations between employment and PROMIS Physical Function scores and number of orthopedic surgeries, better physical function, and fewer surgeries being associated with higher rates of full-time employment. Multivariate analysis also showed an association between WOMAC Pain score and heavy activity, those with worse pain scores being more likely to be in heavy/very heavy work roles than in light/sedentary roles, which may be due to heavy work roles causing greater pain. However, the opposite association might also have been anticipated, those with high levels of pain being unable to undertake roles with high levels of activity.

The bivariate analysis identified additional relationships that were not observed in the multivariate analysis: work status was significantly related to study country, history of osteoarthritis, age at first fracture, number of fractures, lower extremity fractures and traumatic fractures, use of assistive devices, TUG time, PROMIS Physical Function score, WOMAC Pain, Physical Function, and total scores, EQ-5D utility values, and receiving disability payments. Employment description was significantly related to age, history of osteoarthritis, age at first fracture, and receiving disability payments. Activity level was significantly related to utility values. Several of these significant variables were not eligible for inclusion in the multivariate models because of multicollinearity, conceptual overlap of variables, or missing data, but should not be discounted as potential influencers of work productivity.

Until recently, the only treatment for XLH has been supplementation with oral phosphate and active vitamin D. Burosumab is a fully human monoclonal antibody against FGF23 that first became available in 2018.^31–34^ The efficacy and safety of burosumab in adults have been demonstrated in a phase 3 study: serum phosphate concentrations increased to within the normal range and improvements were seen in pain, stiffness, physical function, fatigue, and ambulatory function.^10^^,^^35–37^ As burosumab has been shown to reduce rickets severity and improve growth in children,^38^ it has been hypothesized that burosumab may also reduce the number of orthopedic surgeries through improvements in skeletal alignment.^39^ The association between burosumab treatment and productivity outcomes was assessed in bivariate analysis but results were non-significant or inconclusive and are potentially affected by confounders and sample size issues. Burosumab treatment was not included in multivariate analysis because of conceptual overlap with PROs; previous studies have demonstrated improvements in PROs in subjects receiving burosumab treatment.^10^ Furthermore, the retrospective data were insufficiently detailed in terms of treatment duration, recency of treatment, and treatment gaps to warrant a more thorough investigation. This will be considered in future studies once the XLH-DMP study has accumulated sufficient prospective longitudinal data.

This is a valuable analysis from the patient perspective because it expands understanding of the burden of XLH beyond clinical endpoints to outcomes that are meaningful for patients’ lives. Here, we learn that adults face significant challenges in the workplace because of the complexities of XLH, and are more likely to be unemployed, less likely to be employed full-time, and more likely to be claiming disability payments. This growing picture of the burden of XLH helps legitimize the challenges faced by adults with XLH.

There are several limitations to this study. The study was designed to investigate the association between patient characteristics and work productivity; causality was not assessed and should not be inferred when interpreting the results. Although the XLH-DMP had few exclusion criteria, the possibility of selection bias cannot be excluded. In addition, diversity and inclusivity were not explicitly assessed in recruitment to the program. The study population was predominantly from the US (80.8%, with the remaining 19.2% from Brazil, Canada, and Chile), which may have different employment profiles from other countries, limiting the generalizability of the results outside the US. Patients were not guided on how to categorize the activity level associated with work and there may have been cultural differences in responses across study countries. The XLH-DMP does not include any PROs specifically relating to mental health; thus, interaction between mental health and work productivity in subjects with XLH could not be examined in this analysis. WOMAC Index scores were mapped to EQ-5D utility values using a published algorithm, based on the EQ-5D-3L value set for England.^22^ This algorithm has been previously used in XLH,^40^ has been externally validated, and was found to more accurately capture the characteristics of EQ-5D distribution than other algorithms.^22^^,^^41^ However, the analysis may have been more robust if mapping algorithms based on country-specific value sets were available. Similarly, PROMIS Physical Function score items are calibrated based on a US population, which may limit applicability to other settings.^21^ However, reference data for other countries were not identified.

Selected variables were excluded from multivariate models in the current analysis because of multicollinearity, small sample size, and conceptual overlap; thus, possible significant relationships may not have been identified. Only complete case datasets were used in the multivariate models, so the sample size was smaller than in the bivariate analysis; this may contribute to the lack of significance in multivariate models. Given this, alternative methods for variable selection could be explored in future studies. Finally, the sample size was reduced by excluding patients who joined the study during the COVID-19 pandemic but this was considered preferable to introducing possible confounding. As further data are collected in the XLH-DMP study, more detailed analysis is likely to become possible. Notably, this is the first study to collect granular WOMAC Index data alongside data on work productivity, which may allow further insight into the physical aspects of XLH that have greatest impact on work productivity and the impact of treatment on these outcomes.

## Conclusion

Full-time employment levels are low in adults of working age with XLH and unemployment and disability payment rates are high, suggesting that XLH has a substantial impact on work productivity. Worse physical function and a greater number of orthopedic surgeries are associated with lower work productivity. Worse pain, higher number of orthopedic surgeries, and younger age are associated with heavier work roles, but causality was not specifically investigated.

## Supplementary Material

XLH-DMP_work_productivity_suppl_mat_draft_12_23MAY24_ziae102

## Data Availability

Data that underlie the results reported in this article may be requested. Kyowa Kirin International and Ultragenyx Pharmaceutical will review requests individually to determine whether requests are legitimate, relevant and meet sound scientific principles, and are within the scope of the participants’ informed consent.
