# Supplementary material for: Association between work productivity and characteristics of adults with X-linked hypophosphatemia: an analysis of the XLH disease monitoring program
Source: JBMR Plus. 2024 Jul 29;8(11):ziae102. doi: 10.1093/jbmrpl/ziae102 (PMC11512684; doi:10.1093/jbmrpl/ziae102)

Supplementary materials

**Supplementary Table 1 Bivariate relationships between work productivity and patient demographics (n=281)**

| Patient characteristic | | Work status | | | | Employment description | | | | | Activity level associated with work | | | |
| --- | --- | --- | --- | --- | --- | --- | --- | --- | --- | --- | --- | --- | --- | --- |
|  |  | **Not employed** | **Full-time** | **Part-time** | **P value** | **Full-time outside of home** | **Full-time at home** | **Part-time outside of home** | **Part time at home** | **P value** | **Light or sedentary** | **Medium** | **Heavy or very heavy** | **P value** |
| **n** | | **88** | **150** | **43** |  | **135** | **15** | **30** | **13** |  | **115** | **65** | **13** |  |
| Age (years), mean ± SD (n=281)^a^ | | 40.6 ± 12.8 | 39.9± 11.3 | 38.0 ± 14.5 | 0.532 | 39.9 ± 11.4 | 40.2 ± 10.7 | 34.7 ± 14.6 | 45.7± 11.4 | **0.040** | 41.2 ± 12.0 | 36.7± 11.9 | 38.7± 11.3 | 0.050^c^ |
| Sex, n (%) (n=281)^b^ | Female | 72  (81.8) | 106 (70.7) | 34 (79.1) | 0.130 | 93  (68.9) | 13  (86.7) | 24  (80.0) | 10  (76.9) | 0.338 | 86  (74.8) | 44  (67.7) | 10  (76.9) | 0.554 |
|  | Male | 16  (18.2) | 44  (29.3) | 9  (20.9) |  | 42  (31.1) | 2  (13.3) | 6  (20.0) | 3  (23.1) |  | 29  (25.2) | 21  (32.3) | 3  (23.1) |  |
| Country, n (%) (n=281)^b^ | Brazil | 13  (14.8) | 11  (7.3) | 0 | **0.019** | 10  (7.4) | 1  (6.7) | 0 | 0 | 0.105 | 10  (8.7) | 1  (1.5) | 0 | 0.334 |
|  | Canada | 3  (3.4) | 13  (8.7) | 4  (9.3) |  | 9  (6.7) | 4  (26.7) | 2  (6.7) | 2  (15.4) |  | 10  (8.7) | 5  (7.7) | 2  (15.4) |  |
|  | Chile | 2  (2.3) | 4  (2.7) | 4  (9.3) |  | 4  (3.0) | 0 | 3  (10.0) | 1  (7.7) |  | 6  (5.2) | 2  (3.1) | 0 |  |
|  | USA | 70  (79.5) | 122 (81.3) | 35 (81.4) |  | 112  (83.0) | 10  (66.7) | 25  (83.3) | 10  (76.9) |  | 89  (77.4) | 57  (87.7) | 11  (84.6) |  |

^a^p values calculated using general linear model for unbalanced ANOVA comparing continuous variables across work productivity variables

^b^p value calculated using chi-square comparing categorical variables across work productivity variables

**Bold** indicates significant (p<0.05)

**Supplementary Table 2 Bivariate relationships between work productivity and XLH characteristics (n=281)**

| Patient characteristic | | | Work status | | | | Employment description | | | | | Activity level associated with work | | | |
| --- | --- | --- | --- | --- | --- | --- | --- | --- | --- | --- | --- | --- | --- | --- | --- |
|  |  |  | **Not employed** | **Full-time** | **Part-time** | **P value** | **Full-time outside of home** | **Full-time at home** | **Part-time outside of home** | **Part time at home** | **P value** | **Light or sedentary** | **Medium** | **Heavy or very heavy** | **P value** |
|  |  |  | **88** | **150** | **43** |  | **135** | **15** | **30** | **13** |  | **115** | **65** | **13** |  |
| Age at XLH diagnosis, mean ± SD (n=281)^a^ | | | 6.0 ± 9.5 | 8.6 ± 14.7 | 7.8 ± 12.6 | 0.329 | 9.0 ± 15.0 | 4.7 ± 11.8 | 6.4 ± 11.8 | 11.2 ± 14.0 | 0.500 | 8.0 ± 13.7 | 7.6 ± 13.4 | 16.1 ± 20.6 | 0.130 |
| Family members with XLH, n (%) (n=281)^b^ | | Yes | 61  (69.3) | 118 (78.7) | 36  (83.7) | 0.125 | 104 (77.0) | 14 (93.3) | 25  (83.3) | 11  (84.6) | 0.430 | 88  (76.5) | 55  (84.6) | 11 (84.6) | 0.389 |
|  |  | NR | 27  (30.7) | 32 (21.3) | 7  (16.3) |  | 31 (23.0) | 1  (6.7) | 5  (16.7) | 2  (15.4) |  | 27  (23.5) | 10  (15.4) | 2 (15.4) |  |
| Medical history | | | | | | | | | | | | | | | |
| Osteoarthritis, n (%) (n=281)^b^ | | Yes | 61  (69.3) | 80  (53.3) | 20  (46.5) | **0.017** | 70 (51.9) | 10  (66.7) | 10  (33.3) | 10  (76.9) | **0.033** | 65  (56.5) | 27  (41.5) | 8 (61.5) | 0.119 |
|  |  | NR | 27  (30.7) | 70 (46.7) | 23  (53.5) |  | 65 (48.1) | 5  (33.3) | 20  (66.7) | 3  (23.1) |  | 50  (43.5) | 38  (58.5) | 5 (38.5) |  |
| Enthesopathy/ bone spurs/ osteophytes, n (%) (n=281)^b^ | | Yes | 50  (56.8) | 80 (53.3) | 20  (46.5) | 0.540 | 68 (50.4) | 12 (80.0) | 13  (43.3) | 7  (53.8) | 0.123 | 56  (48.7) | 35  (53.8) | 9 (69.2) | 0.344 |
|  |  | NR | 38  (43.2) | 70 (46.7) | 23  (53.5) |  | 67 (49.6) | 3  (20.0) | 17  (56.7) | 6  (46.2) |  | 59  (51.3) | 30  (46.2) | 4 (30.8) |  |
| Hyper-parathyroidism, n (%) (n=281)^b^ | | Yes | 18  (20.5) | 41 (27.3) | 10  (23.3) | 0.481 | 38 (28.1) | 3  (20.0) | 7  (23.3) | 3  (23.1) | 0.862 | 33  (28.7) | 14  (21.5) | 4 (30.8) | 0.541 |
|  |  | NR | 70  (79.5) | 109 (72.7) | 33  (76.7) |  | 97 (71.9) | 12 (80.0) | 23  (76.7) | 10  (76.9) |  | 82  (71.3) | 51  (78.5) | 9 (69.2) |  |
| Nephrocalcinosis, n (%) (n=281)^b^ | | Yes | 13  (14.8) | 27  (18.0) | 9  (20.9) | 0.660 | 25 (18.5) | 2  (13.3) | 8  (26.7) | 1  (7.7) | 0.461 | 21  (18.3) | 14  (21.5) | 1  (7.7) | 0.497 |
|  |  | NR | 75  (85.2) | 123  (82.0) | 34  (79.1) |  | 110 (81.5) | 13  (86.7) | 22  (73.3) | 12  (92.3) |  | 94  (81.7) | 51  (78.5) | 12  (92.3) |  |
| Number of orthopedic surgeries per subject, mean ± SD (n=280)^a^ | | | 5.7 ± 5.5 | 2.9 ± 3.3 | 3.3 ± 6.1 | **<0.001** | 2.9 ± 3.4 | 2.3 ± 2.5 | 3.5 ± 6.8 | 2.8 ± 4.1 | 0.823 | 3.4 ± 4.7 | 1.9 ± 2.3 | 4.2 ± 4.0 | **0.024** |
| Age at first orthopedic surgery, years, mean ± SD (n=202)^c^ | | | 14.8 ± 9.7 | 14.9 ± 10.5 | 16.7 ± 10.5 | 0.688 | 14.9 ± 10.5 | 15.5 ± 10.8 | 15.8 ± 9.3 | 18.9 ± 13.6 | 0.766 | 15.3 ± 10.6 | 15.0 ± 10.3 | 16.6 ± 11.6 | 0.929 |
| Time from most recent orthopedic surgery to DMP enrolment, years, mean ± SD (n=202)^c^ | | | 13.8 ± 12.6 | 16.2 ± 12.3 | 14.1 ± 12.7 | 0.433 | 16.0 ± 12.4 | 17.1 ± 12.4 | 13.8 ± 12.9 | 14.8 ± 13.0 | 0.872 | 15.9 ± 12.9 | 16.7 ± 12.2 | 9.6 ± 5.8 | 0.332 |
| Burosumab treatment at study entry, n (%) (n=281)^b c^ | Yes | | 46  (52.3) | 66  (44.0) | 20  (46.5) | 0.466 | 63  (46.7) | 3  (20.0) | 11  (36.7) | 9  (69.2) | **0.049** | 53  (46.1) | 28  (43.1) | 5  (38.5) | 0.834 |
|  | No | | 42  (47.7) | 84  (56.0) | 23  (53.5) |  | 72  (53.3) | 12  (80.0) | 19  (63.3) | 4  (30.8) |  | 62  (53.9) | 37  (56.9) | 8  (61.5) |  |
| Exposure to burosumab, n (%) (n=281)^b c^ | Yes | | 65  (73.9) | 108  (72.0) | 33  (76.7) | 0.817 | 97  (71.9) | 11  (73.3) | 22  (73.3) | 11  (84.6) | 0.805 | 80  (69.6) | 52  (80.0) | 9  (69.2) | 0.301 |
|  | No | | 23  (26.1) | 42  (28.0) | 10  (23.3) |  | 38  (28.1) | 4  (26.7) | 8  (26.7) | 2  (15.4) |  | 35  (30.4) | 13  (20.0) | 4  (30.8) |  |
| Fracture/pseudofracture history, mean ± SD | | | | | | | | | | | | | | | |
| Number of fractures/ pseudofractures (n=277)^a, c^ | | | 3.9 ± 9.6 | 1.2 ± 2.3 | 1.3 ± 2.7 | **0.002** | 1.2 ± 2.2 | 1.7 ± 3.0 | 1.4 ± 3.0 | 1.2 ± 2.0 | 0.852 | 1.3 ± 2.4 | 0.9 ± 2.2 | 2.4 ± 2.6 | 0.120 |
| Age at first fracture/ pseudofracture,years (n=137)^a, c^ | | | 21.5 ± 12.1 | 27.6 ± 13.9 | 24.7 ±15.8 | **0.043** | 28.2 ± 14.1 | 24.3 ± 12.4 | 16.7 ± 8.6 | 40.9 ± 14.7 | **0.010** | 26.5 ± 13.3 | 29.1 ± 17.4 | 26.5 ± 12.1 | 0.778 |
| Number of lower extremity fractures/pseudofractures (n=278)^a, c^ | | | 3.0 ± 7.9 | 0.9 ± 1.7 | 1.1 ± 2.4 | **0.004** | 0.9 ± 1.8 | 0.9 ± 1.0 | 1.2 ± 2.6 | 1.0 ± 1.8 | 0.948 | 1.1 ± 2.0 | 0.7 ± 1.3 | 1.9 ± 2.5 | 0.070 |
| Number of non-traumatic/ pseudofractures (n=277)^a^ | | | 3.0 ± 8.7 | 0.9 ± 1.9 | 0.7 ± 1.3 | **0.006** | 0.8 ± 1.7 | 1.2 ± 3.1 | 0.6 ± 1.2 | 0.8 ± 1.5 | 0.756 | 0.8 ± 1.8 | 0.6 ± 1.3 | 1.7 ± 2.6 | 0.097^d^ |
| Number of traumatic fractures (n=280)^a^ | | | 0.9 ± 1.7 | 0.4 ± 1.1 | 0.7 ± 2.3 | **0.034** | 0.4 ± 1.1 | 0.5 ± 0.8 | 0.8 ± 2.7 | 0.3 ± 0.6 | 0.409 | 0.5 ± 1.5 | 0.4 ± 1.4 | 0.7 ± 1.2 | 0.716 |
| Time since first fracture/ pseudofracture to DMP enrolment, years (n=137)^c^ | | | 21.0 ± 14.5 | 15.5 ± 11.9 | 19.6 ± 12.7 | 0.064 | 14.7 ± 11.7 | 20.5 ± 12.8 | 22.4 ± 12.7 | 14.0 ± 11.9 | 0.182 | 18.4 ± 12.9 | 10.8 ± 9.6 | 15.4 ± 8.4 | 0.055 |

^a^p values calculated using general linear model for unbalanced ANOVA comparing continuous variables across work productivity variables

^b^p value calculated using chi-square comparing categorical variables across work productivity variables

^c^Not considered for inclusion in the multinomial logistic regression model because of issues of multicollinearity, conceptual overlap of fracture variables and PRO variables, or missing data

NR, not recorded

**Bold** indicates significant (p<0.05)

**Supplementary Table 3 Bivariate relationships between work productivity and functional/patient-reported outcomes (n=281)**

| Patient characteristic | | Work status | | | | Employment description | | | | | Activity level associated with work | | | |
| --- | --- | --- | --- | --- | --- | --- | --- | --- | --- | --- | --- | --- | --- | --- |
|  |  | **Not employed** | **Full-time** | **Part-time** | **P value** | **Full-time outside of home** | **Full-time at home** | **Part-time outside of home** | **Part time at home** | **P value** | **Light or sedentary** | **Medium** | **Heavy or very heavy** | **P value** |
| n | | **88** | **150** | **43** |  | **135** | **15** | **30** | **13** |  | **115** | **65** | **13** |  |
| Use of assistive devices, n (%) (n=264)^a^ | Yes | 9  (10.2) | 2  (1.3) | 2  (4.7) | **0.005** | 2  (1.5) | 0 | 2  (6.7) | 0 | 0.305 | 3  (2.6) | 1  (1.5) | 0 | 0.768 |
|  | No | 71  (80.7) | 140 (93.3) | 40 (93.0) |  | 128  (94.8) | 12  (80.0) | 28  (93.3) | 12  (92.3) |  | 107  (93.0) | 60  (92.3) | 13 (100.0) |  |
| Use of orthoses, n (%) (n=264)^a^ | Yes | 6  (6.8) | 9  (6.0) | 4  (9.3) | 0.776 | 9  (6.7) | 0 | 1  (3.3) | 3  (23.1) | 0.059^d^ | 10  (8.7) | 3  (4.6) | 0 | 0.349 |
|  | No | 74  (84.1) | 133 (88.7) | 38 (88.4) |  | 121  (89.6) | 12  (80.0) | 29  (96.7) | 9  (69.2) |  | 100  (87.0) | 58  (89.2) | 13 (100.0) |  |
| Pain and/or stiffness,^,^ n (%) (n=281)^a^ | Yes | 80  (90.9) | 137  (91.3) | 39  (90.7) | 0.989 | 123  (91.1) | 14  (93.3) | 26  (86.7) | 13  (100.0) | 0.551 | 103  (89.6) | 61  (93.8) | 12  (92.3) | 0.616 |
|  | NR | 8  (9.1) | 13  (8.7) | 4  (9.3) |  | 12  (8.9) | 1  (6.7) | 4  (13.3) | 0 |  | 12  (10.4) | 4  (6.2) | 1  (7.7) |  |
| Timed Up and Go time (s), mean ± SD (n=264)^b^ | | 12.1 ± 9.2 | 9.3 ± 3.7 | 9.2 ± 2.7 | **0.002** | 9.2 ± 3.7 | 10.2 ± 3.4 | 9.1 ± 2.7 | 9.5 ± 2.7 | 0.820 | 9.4 ± 4.0 | 9.0 ± 2.7 | 9.5 ± 2.5 | 0.735 |
| PROMIS Physical Function score, mean ± SD (n=281)^b^ | | 37.8 ± 8.3 | 44.0 ± 8.3 | 41.3 ± 8.6 | **<0.001** | 44.0 ± 8.4 | 43.6 ± 7.8 | 42.8 ± 8.7 | 38.0 ± 7.6 | 0.101 | 43.2 ± 8.1 | 43.8 ± 8.7 | 42.8 ± 11.0 | 0.849 |
| EQ-5D utility value, mean ± SD (n=277)^b^ | | 0.63 ± 0.3 | 0.74 ± 0.2 | 0.70 ± 0.2 | **0.002** | 0.75 ± 0.2 | 0.69 ± 0.2 | 0.75 ± 0.2 | 0.60 ± 0.2 | 0.081^d^ | 0.75 ± 0.2 | 0.74 ± 0.2 | 0.57 ± 0.3 | **0.013** |
| **WOMAC Index scores, mean ± SD** | | | | | | | | | | | | | | |
| Pain (n=281)^b^ | | 38.8 ± 23.5 | 29.4 ± 21.6 | 29.9 ± 21.6 | **0.006** | 28.8 ± 21.1 | 35.3 ± 25.3 | 27.8 ± 21.9 | 34.6 ± 20.9 | 0.541 | 27.5 ± 20.7 | 30.2 ± 20.5 | 44.2 ± 28.9 | **0.027** |
| Stiffness (n=280)^b^ | | 45.2 ± 24.1 | 42.2 ± 24.0 | 43.3 ± 29.2 | 0.674 | 41.2 ± 23.5 | 50.8 ± 28.1 | 40.8 ± 31.0 | 49.0 ± 24.7 | 0.396 | 40.6 ± 24.6 | 43.8 ± 25.2 | 51.9 ± 29.7 | 0.264 |
| Physical Function (n=277)^b^ | | 35.0 ± 23.3 | 25.7 ± 21.6 | 28.6 ± 24.1 | **0.009** | 25.3 ± 21.3 | 29.3 ± 24.4 | 25.8 ± 24.4 | 34.5 ± 23.2 | 0.503 | 25.2 ± 21.0 | 25.7 ± 21.9 | 38.8 ± 29.9 | 0.107 |
| Total (n=277)^b, c^ | | 36.7 ± 22.3 | 27.8 ± 21.0 | 30.2 ± 23.2 | **0.011** | 27.3 ± 20.6 | 32.4 ± 24.3 | 27.6 ± 23.6 | 35.7 ± 22.1 | 0.494 | 27.0 ± 20.3 | 28.2 ± 21.2 | 41.0 ± 29.3 | 0.081 |
| **Disability payments** | | | | | | | | | | | | | | |
| Receiving, n (%) (n=281)^a, c^ | Yes | 37  (42.0) | 1  (0.7) | 5  (11.6) | **<0.001** | 0 | 1  (6.7) | 3  (10.0) | 2  (15.4) | **0.001** | 4  (3.5) | 2  (3.1) | 0 | 0.791 |
|  | NR | 51  (58.0) | 149  (99.3) | 38  (88.4) |  | 135 (100.0) | 14  (93.3) | 27  (90.0) | 11  (84.6) |  | 111  (96.5) | 63  (96.9) | 13 (100.0) |  |
| Age at first payment, mean ± SD (n=38)^b, c^ | | 34.7 ± 9.7 | 35.0 | 43.2 ± 9.9 | 0.276 | - | 35.0^d^ | 39.3 ± 12.7 | 47.0 ± 8.6 | 0.678 | 43.2 ± 9.9 | 35.0^d^ | 0 | 0.516 |

^a^p value calculated using chi-square comparing categorical variables across work productivity variables

^b^p values calculated using general linear model for unbalanced ANOVA comparing continuous variables across work productivity variables

^c^Not considered for inclusion in the multinomial logistic regression model because of multicollinearity, conceptual overlap between fracture and PRO variables, or missing data

^d^SD could not be calculated because of small sample size

NR, not recorded

**Bold** indicates significant (p<0.05)

Supplementary Table 4 Binomial logistic regression analysis assessing relationships between work status, employment description, and work activity level with utility values

|  | **Category** | **Comparison** | **n** | **Prob > chi^2^** | **R^2^** | **P value** | **Odds ratio** | **95% CI** |
| --- | --- | --- | --- | --- | --- | --- | --- | --- |
| Work status | Full-time work  Part-time work | vs not employed | 277 | 0.002 | 0.045 | **0.001**  0.151 | 8.14  3.22 | 2.489, 26.601  0.653, 15.909 |
| Employment description | Full-time at home  Part-time outside of home  Part-time at home | vs full-time work outside the home | 189 | 0.120 | 0.030 | 0.264  0.957  **0.020** | 0.27  0.95  0.07 | 0.026, 2.726  0.123, 7.253  0.007, 0.653 |
| Activity level | Heavy or very heavy  Medium | vs light or sedentary | 189 | 0.030 | 0.036 | **0.007**  0.731 | 0.04  0.77 | 0.005, 0.421  0.167, 3.511 |

**Bold** indicates significant (p<0.05)

Supplementary Figure 1 Multinomial logistic regression assessment of the relationships between employment description and patient characteristics (n=184)


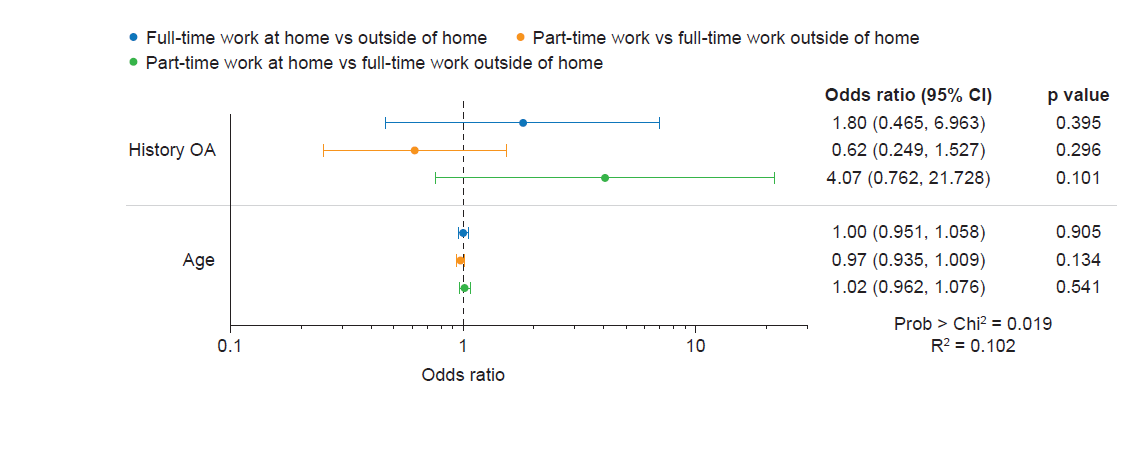


Use of orthoses (yes vs no) is not presented because the sample size was too small for accurate parameter estimation.

OA, osteoarthritis

Supplementary Figure 2 Multinomial logistic regression analysis assessing relationships between work status and utility value (n=258)


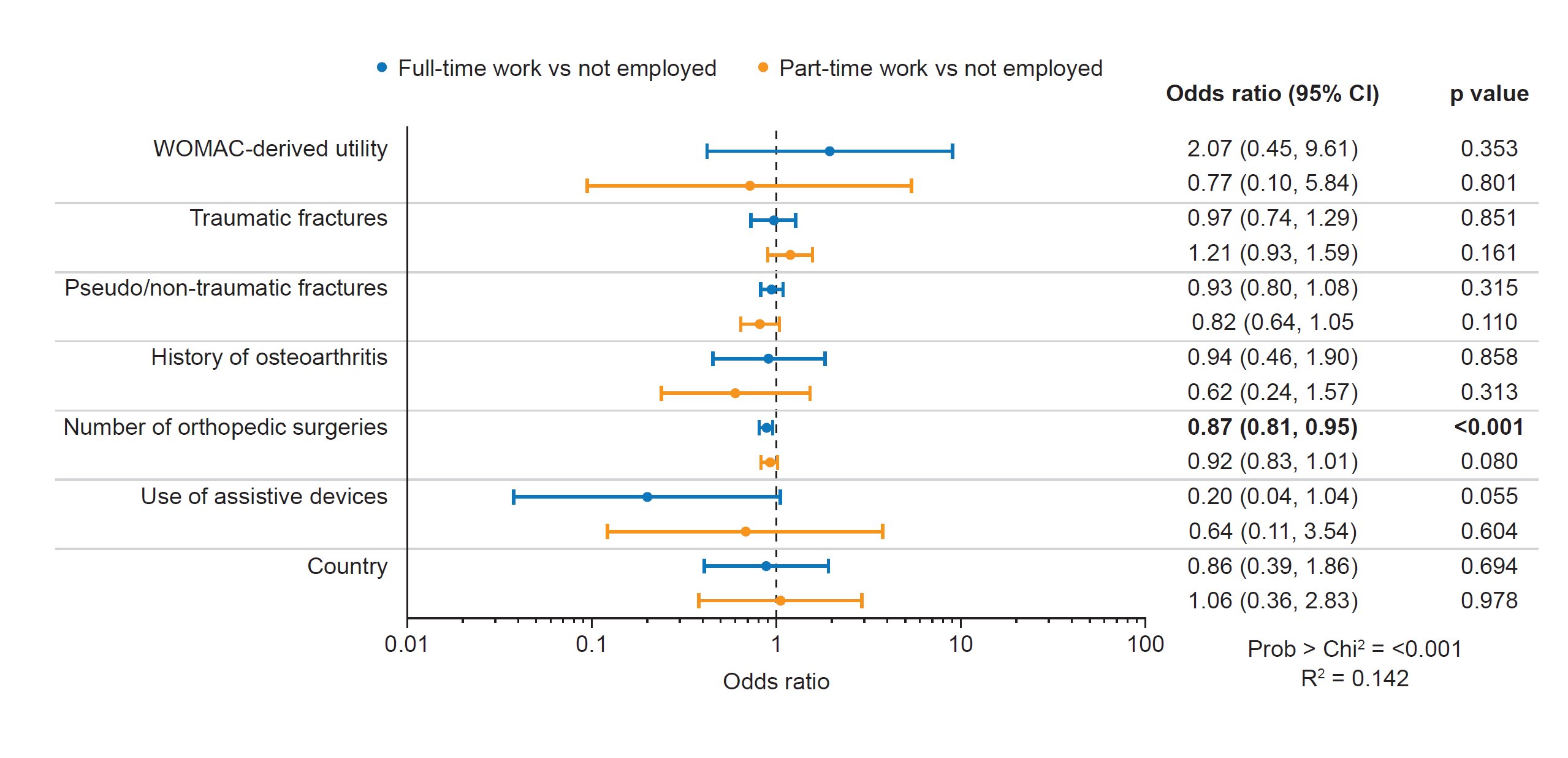


Supplementary Figure 3 Multinomial logistic regression analysis assessing relationships between employment description and utility values (n=181)


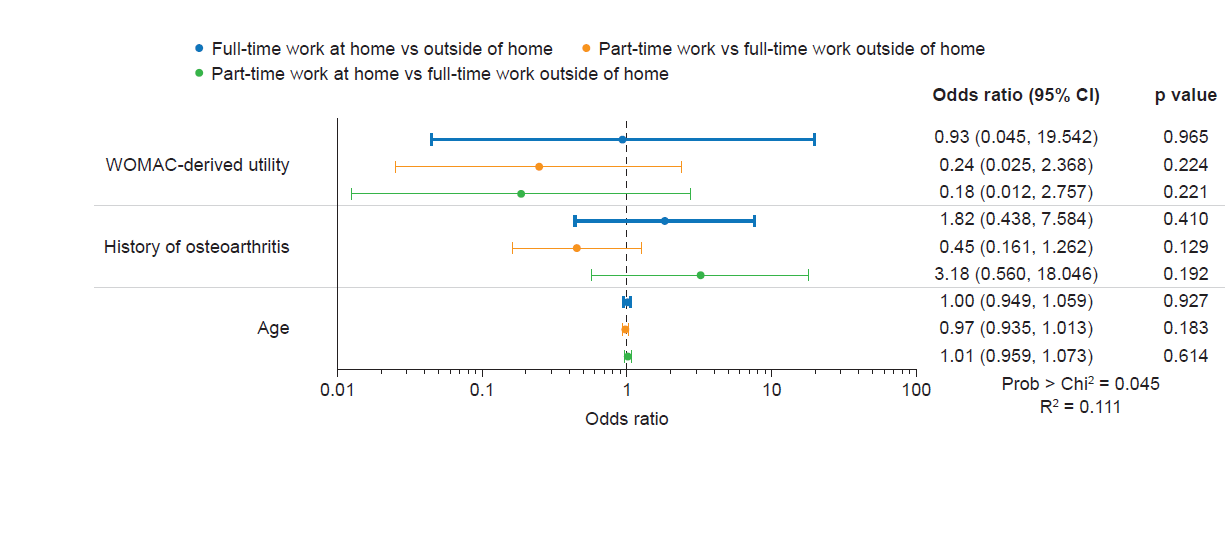


Use of orthoses (yes vs no) is not included in the figure because the sample size was too small for accurate calculation

Supplementary Figure 4 Multinomial logistic regression analysis assessing relationships between work activity level and utility values (n=188)
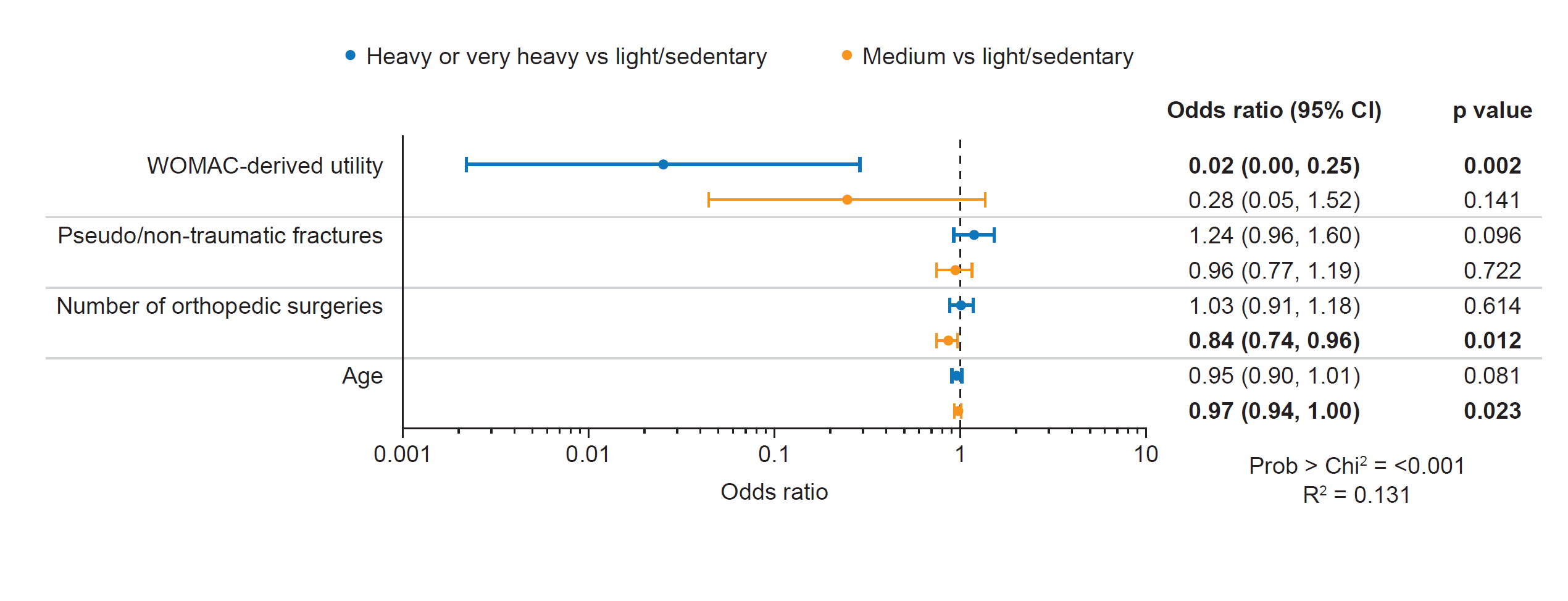

Supplement: XLH-DMP_work_productivity_suppl_mat_draft_12_23MAY24_ziae102 [file xlh-dmp_work_productivity_suppl_mat_draft_12_23may24_ziae102.docx]
